# Daily estradiol and progesterone levels moderate genetic and environmental influences on emotional eating across 45 consecutive days in female twins

**DOI:** 10.1017/S0033291724002770

**Published:** 2024-11

**Authors:** Kelly L. Klump, Megan E. Mikhail, Carolina Anaya, Pamela K. Keel, Kristen M. Culbert, Cheryl L. Sisk, Alexander Johnson, Steven Boker, Micheal C. Neale, S. Alexandra Burt

**Affiliations:** 1 Department of Psychology, Michigan State University, East Lansing, MI, USA; 2 Department of Psychology, Florida State University, Tallahassee, FL, USA; 3 Neuroscience Program, Michigan State University, East Lansing, MI, USA; 4 Department of Psychology, University of Virginia, Charlottesville, VA, USA; 5 Departments of Psychiatry, Human Genetics, and Psychology, Virginia Commonwealth University, Richmond, VA, USA

**Keywords:** binge eating, emotional eating, environmental, estrogen, genetic, progesterone, twins

## Abstract

**Background:**

Past studies indicate daily increases in estrogen across the menstrual cycle protect against binge-eating (BE) phenotypes (e.g. emotional eating), whereas increases in progesterone enhance risk. Two previous studies from our laboratory suggest these associations could be due to differential genomic effects of estrogen and progesterone. However, these prior studies were unable to directly model effects of daily changes in hormones on etiologic risk, instead relying on menstrual cycle phase or mean hormone levels. The current study used newly modified twin models to examine, for the first time, the effects of daily changes in estradiol and progesterone on genetic/environmental influences on emotional eating in our archival twin sample assessed across 45 consecutive days.

**Methods:**

Participants included 468 female twins from the Michigan State University Twin Registry. Daily emotional eating was assessed with the Dutch Eating Behavior Questionnaire, and daily saliva samples were assayed for ovarian hormone levels. Modified genotype × environment interaction models examined daily changes in genetic/environmental effects across hormone levels.

**Results:**

Findings revealed differential effects of daily changes in hormones on etiologic risk, with increasing genetic influences across progesterone levels, and increasing shared environmental influences at the highest estradiol levels. Results were consistent across primary analyses examining all study days and sensitivity analyses within menstrual cycle phases.

**Conclusions:**

Findings are significant in being the first to identify changes in etiologic risk for BE symptoms across daily hormone levels and highlighting novel mechanisms (e.g. hormone threshold effects, regulation of conserved genes) that may contribute to the etiology of BE.

Estrogen and progesterone substantially impact risk for binge-eating (BE) phenotypes across the menstrual cycle in women. Higher estradiol protects against BE (i.e. consumption of a large amount of food in a short period of time with loss of control; American Psychiatric Association, 2022) and emotional eating (i.e. over-eating in response to negative emotions), while higher progesterone antagonizes these effects and lead to increased BE/emotional eating, particularly during high-risk menstrual cycle phases (e.g. midluteal phase; Klump et al., 2013b; Klump et al., 2014). These hormone/BE associations are independent of body mass index (BMI) (Edler, Lipson, & Keel, 2007; Klump et al., 2013a; Klump et al., 2013b), negative affect (Edler et al., 2007; Klump et al., 2013a, 2013b; Racine et al., 2013), dietary restraint (Klump et al., 2013a), weight concerns (Hildebrandt et al., 2015), and impulsive traits (Racine et al., 2013), and have been observed in community samples (Edler et al., 2007; Klump et al., 2013b) and women with clinical BE (Edler et al., 2007; Klump et al., 2014). Similar hormone effects are also observed for food intake and overconsumption of highly palatable food (e.g. high fat/sugar foods) in experimental animal studies (Asarian & Geary, 2013; Klump, Culbert, & Sisk, 2017). The confluence of findings across BE symptoms, key moderators/covariates, and species speaks to the robust nature of ovarian hormone effects on BE.

However, much less is known about *how* ovarian hormones exert these phenotypic effects. Initial hypotheses have focused on genomic effects within the central nervous system (CNS), as ovarian hormones directly regulate gene transcription and protein synthesis in key neural systems for BE (e.g. dopamine, serotonin; Del Río et al., 2018). Despite this theoretical framework, to date, no study has directly examined gene regulation as a mechanism for hormone effects on emotional eating/BE. The absence of such data reflects difficulties measuring gene transcription/expression in humans and the lack of identified risk genes for BE.

One indirect method for examining genomic effects is to investigate changes in heritability across ovarian hormone levels. Because changes in heritability reflect changes in the influence of genetic factors, this approach provides a straightforward way to rule in (or out) changes in genetic risk (and potentially gene expression) for BE. In addition, changes in heritability index genetic risk at the latent, aggregate level, and thus, analyses are not contingent upon selection of particular genes/polygenic risk scores. Although these analyses cannot definitively determine if changes in gene expression underlie effects, they provide important initial tests of the importance of genetic factors in hormone effects on BE.

We previously published a twin study examining differences in the heritability of emotional eating between women with lower *v.* higher mean ovarian hormone levels (Klump et al., 2016). Although daily data (across 45 consecutive days) were collected in this study, no existing twin models were able to examine repeated measures of both hormones and emotional eating or how daily changes in ovarian hormones impacted changes in emotional eating. Thus, we averaged hormone levels and emotional eating scores across the 45 days and used median splits to compare genetic/environmental influences between women who had lower *v.* higher trait-levels of hormones. The heritability of emotional eating was significantly greater in twins with higher mean progesterone levels (61%) as compared to twins with lower levels (37%) (Klump et al., 2016). By contrast, shared environmental influences (i.e. environmental factors that are common to twins reared in the same family) on emotional eating were significantly greater in twins who had higher (25%) *v.* lower (0%) estradiol levels. These findings mapped onto a previous analysis across menstrual cycle phase in the same sample (Klump et al., 2015), where stronger genetic influences on emotional eating scores (averaged within phase) were observed during the post-ovulatory phase of the cycle (when progesterone levels/BE risk are high), and stronger shared environmental effects were observed during the pre-ovulatory phase (when estradiol levels increase and BE risk is low).

Despite the promise of these initial results, large gaps remain in our understanding of hormone effects on etiologic risk. Most notably, it remains unclear how daily changes in ovarian hormones impact genetic/environmental influences on emotional eating. Our study of menstrual cycle phase (Klump et al., 2015) did not examine ovarian hormone levels, making it impossible to confirm that differences in genetic/environmental influences across cycle phase were driven by ovarian hormones. Conversely, our analyses of mean hormone levels (Klump et al., 2016) focused on trait-levels that provide information about *who* exhibits stronger genetic (e.g. women with higher trait progesterone) *v.* shared environmental influences (i.e. women with higher trait estradiol), but were unable to determine how daily changes in hormones impact day-to-day changes in BE etiologic risk. Ovarian hormones change dynamically across days and the menstrual cycle, and it is these daily changes that most substantially impact phenotypic risk for BE (rather than mean hormone levels; Klump et al., 2013b, 2014). It is therefore critical to determine whether daily fluctuations in ovarian hormones are associated with changes in genetic/environmental influences on BE.

As noted above, we were unable to examine daily hormone effects in previous work due to the lack of available twin models to analyze repeated measures within a gene × hormone/environment framework. In fact, to our knowledge, no twin studies have examined changes in gene × hormone/environment interactions across days or other repeated measures, likely due to the resources needed to collect these data in genetically informed samples and the lack of analytic tools for probing changing gene × environment effects. Consequently, in the current study, we capitalized on the availability of our unique archival data and developed a novel modification of existing twin models to examine the effects of daily hormone levels on genetic/environmental influences on daily emotional eating scores in our female twins assessed across 45 consecutive days. These archival data are highly unique in their scope (over 26 000 daily hormone and emotional eating values) and ability to model both linear and non-linear effects of hormones on etiologic risk. Our past study of hormones was limited to examining linear effects only due to the use of dichotomized hormone values (Klump et al., 2016). Because hormones fluctuate in a very non-linear fashion in women (e.g. increases and then decreases across the cycle), dichotomized levels provide an incomplete and potentially inaccurate picture of the nature/impact of hormones on BE risk, particularly if non-linearity is the norm rather than the exception. Examining linear and non-linear trajectories of etiologic risk at the daily level is critically important for generating an accurate and complete picture of ovarian hormone influences on BE.

## Method

### Participants

Analyses included 468 participants (ages 15–25; *M_age_* = 17.83, s.d. = 1.79) from same-sex female pairs participating in the *Twin Study of Hormones and Behavior Across the Menstrual Cycle* (HBMC; Klump et al., 2013b). Participants were recruited through the Michigan State University Twin Registry (MSUTR), which identifies twins through birth records (see Burt & Klump, 2019). The MSUTR has similar response rates to other twin registries, and MSUTR twins are demographically representative of Michigan (Burt & Klump, 2019). Eligibility criteria for HBMC included: (1) menstruation every 22–32 days for the past 6 months; (2) no hormonal contraceptive use in the past 3 months; (3) no psychotropic or steroid medications in the past 4 weeks; (4) no pregnancy or lactation in the past 6 months; and (5) no history of genetic/medical conditions known to influence hormone functioning or appetite/weight (e.g. polycystic ovary syndrome, Turner syndrome) (Klump et al., 2013b).

Twins who exclusively experienced anovulatory or flat cycles were excluded from analyses (*N* = 43 participants, 8.4% of original sample), as the aims of the study were to capture how dynamic, daily changes in ovarian hormones impact changes in genetic/environmental influences on emotional eating. If participants experienced one ovulatory and one anovulatory cycle, data from their ovulatory cycle were included. Excluded participants did not significantly differ from included participants on mean emotional eating scores (*p* = 0.239, *d* = 0.19). Moreover, HBMC participants did not differ from other MSUTR twins on disordered eating symptoms (e.g. emotional eating, body dissatisfaction; *d*s = 0.02–0.18) (Klump et al., 2013b) and were representative of the recruitment region in terms of race and ethnicity (see Table 1).

**Table 1.** Participant demographic characteristics (*N* = 468 twins)

Participant Demographics	Mean (s.d.) or % of Sample (*N*)
Age	17.83 (1.79) (range = 15–25)
Sex	
Female (sex assigned on birth certificate)	100% (468)
Zygosity	
Twins from complete monozygotic (MZ) pairs	252 (53.8%)
Twins from complete dizygotic (DZ) pairs	200 (42.7%)
Twins without cotwin data	16 (3.4%)
Racial identity	
White	78.6% (368)
Black/African American	10.9% (51)
Asian/Asian American	0.4% (2)
American Indian/Alaska Native	0.4% (2)
More than one race	4.7% (22)
Other/unknown	4.9% (23)
Ethnicity	
Hispanic/Latinx	42 (9.0%)
Parental income	
Under $ 20 000	24 (5.1%)
$ 20 000–$ 40 000	64 (13.7%)
$ 40 000–$ 60 000	109 (23.3%)
$ 60 000–$ 100 000	153 (32.7%)
Over $ 100 000	98 (20.9%)
Unknown/not reported	20 (4.3%)

### Procedure

As described in Klump et al. (2013b), participants provided saliva samples for ovarian hormones each morning (within 30 min of waking) and emotional eating ratings each evening (after 5:00 pm) for 45 consecutive days. This approach ensured hormone measures for a given day preceded behavioral ratings. Saliva samples were retrieved from participants and study eligibility and adherence were reconfirmed during three in-person assessments at the beginning, midpoint, and end of data collection. Between assessments, staff contacted participants once per week to answer questions and confirm protocol adherence. Dropout (3%) and missing data (⩽6%) were minimal, and only a small number (3%) of participants became ineligible due to pregnancy or medication use (Klump et al., 2013b).

### Measures

#### Emotional eating

We used the 13-item Dutch Eating Behavior Questionnaire emotional eating scale (DEBQ; van Strien, Frijters, Bergers, & Defares, 1986) modified with permission to refer to that day. DEBQ emotional eating scores correlate strongly with other BE measures (e.g. van Strien, 1996) and palatable food consumption in laboratory settings (van Strien, 2000). The DEBQ emotional eating scale shows excellent internal consistency in our sample (average *α* = 0.90). Similar to previous population-based studies (e.g. Nagl, Hilbert, de Zwaan, Braehler, & Kersting, 2016), emotional eating varied substantially in our sample (range = 0–3.69; see online Supplementary Table S1 in Supplemental Material), and distinguished between participants with and without BE episodes assessed via the Structured Clinical Interview for DSM (First, Spitzer, Gibbon, & Williams, 1996) (*d* = 0.84, *p* < 0.001). Emotional eating scores for a given day were prorated if ⩽10% of items were missing and marked as missing otherwise.

#### Ovarian hormones

Participants provided saliva samples using published methods (Klump et al., 2013b; Klump, Keel, Culbert, & Edler, 2008). Serum measures of hormones were not feasible for our daily study design that involved collecting hormone samples across 45 consecutive days. Saliva collection is a non-invasive method for repeated sampling schedules that is associated with higher compliance and more robust hormone-behavior associations than other methods (e.g. bloodspots; see Edler et al., 2007). Our use of multiple hormone measures per participant (> 10 000 observations across participants – see online Supplementary Table S1 in Supplemental Materials) also helps allay concerns that may arise when using only one or few hormone measurements. Saliva samples were assayed using specialized enzyme immunoassay kits (Salimetrics, LLC) that show excellent reliability (intra- and inter-assay coefficients of variation: estradiol = 7.1 and 7.5%; progesterone = 6.2 and 7.6%), specificity (determined by interpolating the mean optical density minus 2 s.d. of 10–20 replicates at the 0 pg/ml level; estradiol = 0.10 pg/ml; progesterone = 5 pg/ml), and method accuracy (measured via spike recovery and linearity; estradiol = 104.2 and 99.4%; progesterone = 99.6 and 91.8%). To optimize resources, all samples collected during key periods of hormonal change (i.e. mid-follicular through premenstrual phases) were assayed, whereas samples from every other day were assayed when hormone levels were low and stable (i.e. menstrual bleeding, early follicular phase).

Similar to previous research (e.g. Klump et al., 2013a, 2014), five-day rolling averages were calculated for estradiol and progesterone. Rolling averages are standard in hormone-behavior research (Kassam et al., 1996; Waller et al., 1998) because they reduce ‘noise’ introduced through pulsatile hormone release (Gladis & Walsh, 1987) and help account for potential delays in hormone effects on behavior (Eckel, 2011). Hormone rolling averages were calculated if there were ⩾3 days of data within the 5-day window and counted as missing if there were <3 days of data. Hormone levels were in the expected range and followed expected patterns across menstrual cycle phase, with estradiol peaking during ovulation and progesterone peaking during the midluteal phase (see online Supplementary Table S1). Consistent with past research (Klump et al., 2017), mean emotional eating scores were higher during the midluteal phase relative to the ovulatory phase (*p* = 0.008).

### Statistical analyses

All analyses were conducted in Mplus version 8.6 (Muthén & Muthén, 1998-2021) using full information maximum likelihood (FIML) estimation. This estimator treats missing data as missing-at-random and is expected to produce less biased and more consistent estimates than other techniques (e.g. listwise deletion). Our analyses built on the van der Sluis, Posthuma, and Dolan (2012) model for genotype × environment analyses in which cotwins can differ on levels of the moderator, with novel modifications to allow for repeated measures (described below). This model examines differences in additive genetic (A; i.e. genetic influences that sum across genes), shared environmental (C; i.e. environmental factors that increase similarity between co-twins), and nonshared environmental (E; i.e. environmental factors that differentiate co-twins, including measurement error) influences on emotional eating across ovarian hormone levels.

We focused on single moderator models for estradiol and progesterone. Because estradiol and progesterone levels were significantly correlated in our sample (*r*'s = 0.48–0.62 across phase), we modeled the independent effects of each hormone by regressing out levels of the other hormone prior to analyses (e.g. regressed progesterone levels out of estradiol values for estrogen analyses). Ideally, we would have also fit double moderator, estradiol × progesterone models. However, our sample size prohibited us from fitting these models, as simulation studies suggest very large samples are needed for these models (Burt, Clark, Pearson, Klump, & Neiderhiser, 2020; Purcell, 2002). Moreover, we observed non-linear changes in etiologic effects in our models (see below), and double moderator models do not easily allow for these types of non-linear changes.

**Figure 1.**
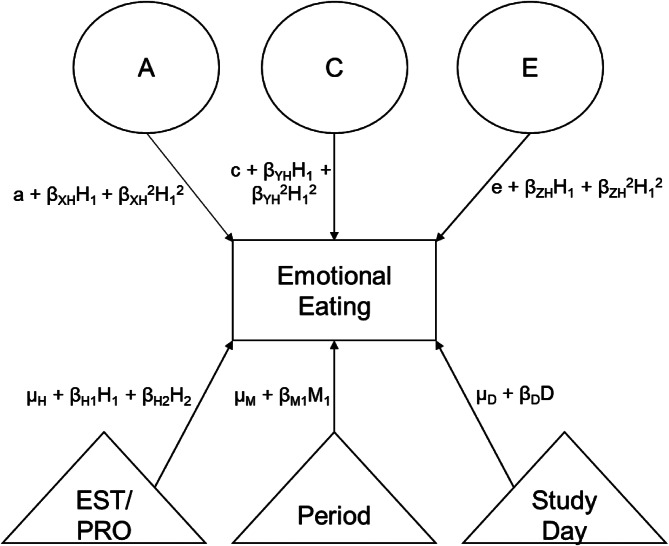
Path Diagram for the Full Twin Daily Moderation Model. EST, estradiol; PRO, progesterone; period, whether the participant experienced menstrual bleeding that day; study day, day of participation in the study; A, additive genetic influences; C, shared environmental influences; E, nonshared environmental influences; *H*_1_ and *H*_2_, hormone levels (estradiol or progesterone) for twin 1 and twin 2 on a given day; *M*_1_, whether twin 1 was menstruating on a given day (coded 0 or 1); D, day of participation; *μ*_H_, *μ*_M_, *μ*_D_, a, c, e, intercepts; *β*_H1_, regression coefficient representing the phenotypic association between twin 1's daily hormone levels and their own emotional eating; *β*_H2_, regression coefficient representing the phenotypic association between twin 2's daily hormone levels and twin 1's emotional eating; *β*_D_, regression coefficient representing the phenotypic association between study day and twin 1's disordered eating; *β*_M1_, regression coefficient representing the phenotypic association between twin 1's menstrual status and their own emotional eating; *β*_XH_, *β*_YH_, *β*_ZH_, coefficients for linear moderation of genetic and environmental influences by hormone levels (estradiol or progesterone); *β*_XH_^2^, *β*_YH_^2^, *β*_ZH_^2^, coefficients for quadratic moderation of genetic and environmental influences by hormone levels (estradiol or progesterone). Analyses within phase exclude the menstrual phase and study day components but are otherwise identical.

Our hormone moderation models (see Fig. 1) included nine parameters of interest: three path coefficients (a, c, e) that capture genetic/environmental influences when hormone levels are lowest, and six moderation coefficients that capture linear (*β*_XH_, *β*_YH_, *β*z_H_) and non-linear/quadratic (*β*_XH_^2^, *β*_YH_^2^, *β*z_H_^2^) increases/decreases in the initial ACE path coefficients as a function of hormone levels. Data were analyzed in ‘wide’ format, wherein each row contained observations for both twins in a family on each day of the study. This allowed for calculation of how genetic/environmental influences on emotional eating changed as a function of each twin's daily hormone level. These analyses used the ‘cluster’ function along with a robust FIML estimator and the Satorra–Bentler scaled change in χ^2^ (Satorra, 2000), which account for clustering of multiple daily observations within families as well as non-normality. To ensure adequate numbers of observations at each level of the moderator, daily hormone values were ‘binned’ into percentiles relative to hormone values across all participants on all days of study participation. We also controlled for whether a participant was menstruating (coded 0 = no, 1 = yes) to account for effects of menstruation-related symptoms (e.g. pain) on emotional eating above and beyond daily hormone levels, and the day of participation (coded day 1 through day 45) to account for reactivity effects across the 45 days (e.g. potential decreases in emotional eating over time due to increased monitoring that could confound hormone effects) (see Data Code and Availability statement below for links to the scripts for these models).

In terms of model-fitting, we first fit the full model with all path estimates and moderators freely estimated. We then fit models that constrained all genetic, shared environmental, and nonshared environmental moderators to zero. We used patterns of results from these models to test nested submodels that individually constrained linear and non-linear moderators to zero. The model that showed a non-significant difference in minus twice the log-likelihood (−2 lnL) between the full and nested model, and minimized Akaike's Information Criterion (AIC), Bayesian Information Criterion (BIC), and sample-size adjusted BIC (SABIC), was chosen as best fitting. If AIC, BIC, and SABIC identified different models as best-fitting, the model that optimized two out of three indices and had a non-significant change in −2 lnL was selected as the best-fitting. Because each model included different datapoints, sample sizes varied across analyses (see online Supplementary Table S1 in Supplemental Material). Following previous recommendations (Purcell, 2002), tables and figures report unstandardized path coefficients and moderator estimates that reflect absolute differences in genetic/environmental influences across the moderators. However, standardized estimates are provided in the text to allow for comparisons with previous gene × hormone findings (Klump et al., 2015, 2016).^†^^1^

## Results

### Estrogen models

**Figure 2.**
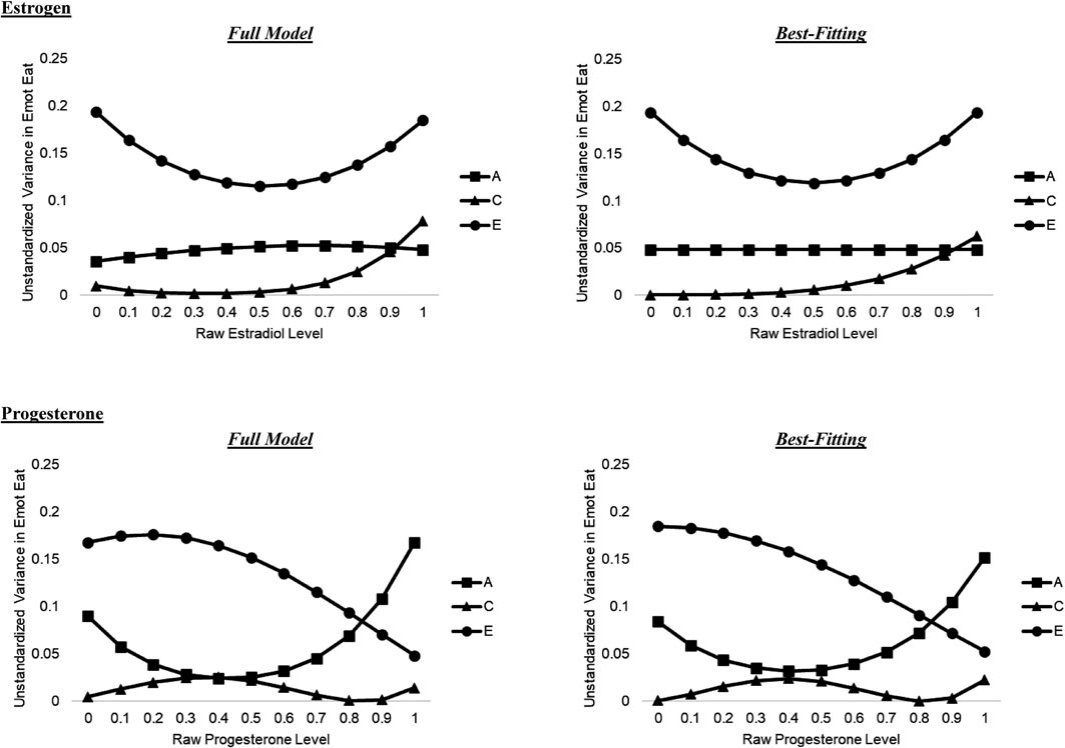
Changes in Genetic and Environmental Influences on Emotional Eating across Daily Ovarian Hormone Levels in the Full and Best-Fitting Models. A, additive genetic influences; C, shared environmental influences; E, nonshared environmental influences; Emot Eat, emotional eating. The *X* axis depicts raw hormone values that were binned into percentiles for analyses.

Results from the full model indicated likely moderation by estradiol levels (see Fig. 2). Changes appeared to be non-linear and most pronounced for shared and nonshared environmental influences. Shared environmental influences were nominal at low and moderate estradiol levels, then increased sharply with increasing estradiol. By contrast, nonshared environmental effects initially decreased and then increased across estradiol levels. Additive genetic influences remained relatively stable and low despite significant daily changes in estradiol.

Model-fitting analyses confirmed these impressions (see Table 2). The model constraining all genetic moderators (i.e. no A mods) provided a better fit to the data (i.e. lower AIC, BIC and SABIC) than the full model, the no moderation model, and the model constraining all shared or nonshared environmental moderators (i.e. no C or E mods). Additional submodel testing identified the best-fitting model as one that constrained all genetic moderators and the linear shared environmental moderator to zero. This model showed a non-significant change in χ^2^ from the full model and the lowest AIC, BIC, and SABIC values. Although the shared environmental estimates were not significant in this model (see Table 3), the better fit of this model suggested that limited power likely constrained our ability to provide precise estimates of these effects. The pattern of changes across estradiol levels resembled the full model (see Fig. 2), with a non-linear increase in shared environmental influences, decreasing and then increasing nonshared environmental influences, and no changes in genetic effects. Standardized parameter estimates indicated stronger shared environmental influences at higher (~21%) *v.* lower (<1%) estradiol levels. Taken together, results suggest daily estrogen levels substantially impact environmental influences on emotional eating but have minimal effects on additive genetic influences.

**Table 2.** Model fit comparisons for estrogen and progesterone models

Models	−2 lnL	Scaling Correction Factor	χ^2^Δ (df)	*p*	AIC	BIC	SABIC
Estrogen Models							
Full model	12 795.78	19.52	–	–	12 829.78	12 941.59	12 887.57
No A mods	12 796.29	20.74	0.05 (2)	0.976	12 826.29	12 924.94	12 877.28
No A quad mod	12 796.02	20.04	0.02 (1)	0.885	12 828.02	12 933.25	12 882.41
No A linear mod	12 796.14	20.15	0.04 (1)	0.845	12 828.14	12 933.38	12 882.53
No A mods, *no C quad mod*	12 799.63	21.24	0.33 (3)	0.953	12 827.63	12 919.71	12 875.22
**No A mods, *no C linear mod***	**12 796.83**	**21.53**	**0.10 (3)**	**0.991**	**12 824.83**	**12 916.91**	**12 872.42**
No C mods	12 803.60	21.00	0.93 (2)	0.628	12 833.60	12 932.26	12 884.60
No C quad mod	12 798.67	19.95	0.23 (1)	0.633	12 830.67	12 935.90	12 885.06
No C linear mod	12 796.76	20.02	0.09 (1)	0.769	12 828.76	12 934.00	12 883.15
No E mods	12 819.76	19.55	1.24 (2)	0.537	12 849.76	12 948.42	12 900.76
No AC mods	12 820.72	21.50	1.91 (4)	0.753	12 846.72	12 932.22	12 890.91
No moderation	12 934.14	18.97	6.74 (6)	0.345	12 956.14	13 028.49	12 993.54
Progesterone Models							
Full model	12 778.81	16.91	–	–	12 812.81	12 924.63	12 870.61
No A mods	12 794.47	17.98	1.76 (2)	0.415	12 824.47	12 923.13	12 875.46
No A quad mod	12 793.98	17.83	7.03 (1)	0.008	12 825.98	12 931.22	12 880.37
No A linear mod^a^	12 793.00	18.02	6.58 (1)	0.010	12 825.00	12 930.24	12 879.39
No C mods	12 798.97	17.84	2.03 (2)	0.363	12 828.97	12 927.63	12 879.96
No C quad mod	12 785.40	17.43	0.76 (1)	0.384	12 817.40	12 922.64	12 871.80
No C linear mod	12 781.95	17.36	0.32 (1)	0.570	12 813.95	12 919.18	12 868.34
No E mods	12 872.71	18.31	14.53 (2)	<0.001	12 902.71	13 001.37	12 953.70
No E quad mod	12 790.72	17.31	1.12 (1)	0.289	12 822.72	12 927.95	12 877.11
**No E linear mod**	**12 780.34**	**17.19**	**0.12 (1)**	**0.727**	**12 812.34**	**12 917.58**	**12 866.74**
No E linear mod, *no C linear mod*	12 787.78	17.59	0.76 (2)	0.685	12 817.78	12 916.43	12 868.77
No moderation	12 952.43	18.11	11.79 (6)	0.067	12 974.43	13 046.78	13 011.83

*Note*. A, additive genetic influences; C, shared environmental influences; E, nonshared environmental influences; quad, quadratic/non-linear effects; −2 lnL, minus twice the log-likelihood; Δχ^2^, the difference in −2 lnL values between the full model and the nested model, which is χ^2^ distributed under the null hypothesis implied by the reduced model; df, degrees of freedom; AIC, Akaike Information Criterion; BIC, Bayesian Information Criterion; SABIC, sample size adjusted Bayesian Information Criterion. Dashes indicate parameters are not applicable. The best-fitting model is bolded.

a The standard Satorra-Bentler Scaled χ^2^ difference test gave a negative value, and thus the alternative ‘strictly positive’ Satorra-Bentler χ^2^ difference test was used.

**Table 3.** Parameter estimates from the full and best-fitting models

Models	a	Linear a mod	Quad a mod	c	Linear c mod	Quad c mod	e	Linear e mod	Quad e mod
Estrogen Models									
Full model	0.19 (−0.10 to 0.48)	0.12 (−1.17 to 1.41)	−0.09 (−1.36 to 1.18)	0.10 (−0.31 to 0.51)	−0.35 (−2.24 to 1.53)	0.53 (−1.14 to 2.21)	**0.44 (0.32–0.56)**	−0.39 (−0.91 to 0.14)	0.38 (−0.18 to 0.93)
Best fitting	**0.22 (0.14–0.30)**	–	–	0.02 (−0.08 to 0.12)	–	0.23 (−0.05 to 0.52)	**0.44 (0.34–0.54)**	−0.38 (−0.79 to 0.04)	0.38 (−0.10 to 0.85)
Progesterone Models									
Full model	**0.30 (0.10–0.50)**	−0.67 (−1.56 to 0.23)	0.78 (−0.23 to 1.79)	0.07 (−0.49 to 0.63)	0.50 (−0.22 to 2.23)	−0.69 (−2.22 to 0.84)	**0.41 (0.27–0.55)**	0.11 (−0.48 to 0.69)	−0.30 (−0.86 to 0.27)
Best fitting	**0.29 (0.11–0.47)**	−0.53 (−1.32 to 0.27)	0.63 (−0.27 to 1.52)	0.03 (−0.46 to 0.53)	0.64 (−0.74 to 2.03)	−0.82 (−2.03 to 0.38)	**0.43 (0.33–0.52)**	–	−0.20 (−0.35 to −0.04)

*Note.* a, additive genetic influences; c, shared environmental influences; e, nonshared environmental influences; mod, moderator; quad, quadratic/non-linear effects. Dashes indicate that the parameter estimate was constrained to be 0. Significant model parameters are bolded, with the 95% CI in parentheses.

Nonetheless, given non-significant moderator estimates, we conducted sensitivity analyses examining the effects of estradiol within menstrual cycle phase (i.e. follicular, ovulatory, midluteal, premenstrual, and menstrual) rather than across all study days. Variations in estrogen's influences by phase could attenuate the magnitude of effects when examining estrogen across all study days. The full models showed increasing shared environmental influences across estradiol levels in all phases (see online Supplementary Fig. S1 in Supplemental Material), and the shared environmental moderators were retained in nearly all models (see online Supplementary Table S2). Effects were particularly pronounced during the ovulatory phase when estradiol levels increase dramatically and reach their peak. The shared environmental moderators were statistically significant in several phases (e.g. the ovulatory phase; see online Supplementary Table S4), and the additive genetic and nonshared environmental moderators could be constrained to zero without a worsening of model fit.

### Progesterone models

Findings for progesterone showed stronger moderation of additive genetic influences, with genetic effects increasing substantially in a non-linear fashion across progesterone levels. By contrast, nonshared environmental influences decreased across progesterone levels. Changes in shared environmental influences were more modest, with these influences appearing strongest at moderate progesterone levels.

Model-fitting confirmed these impressions (see Table 2). The full model provided a better fit to the data (i.e. lower AIC, BIC, and SABIC) than the model constraining all etiologic moderators to zero. χ^2^ change tests for the models constraining just the genetic moderators to zero were statistically significant, suggesting these moderators could not be dropped from the model without significantly worsening fit.

The best-fitting model constrained the linear nonshared environmental moderator but retained all genetic (and shared environmental) moderators (see Table 2). This model evidenced a non-significant change in χ^2^ from the full model and the lowest AIC, BIC, and SABIC values. Moderator estimates were not always statistically significant (see Table 3), but the clear decrement in model fit when genetic parameters were constrained to zero highlights the importance of these factors. The pattern of etiologic effects resembled the full model (see Fig. 2), and standardized parameter estimates showed substantial increases in genetic influences from lower (31%) to higher (67%) progesterone levels. By contrast, nonshared environmental influences decreased (69–23%) across progesterone levels. Changes for shared environmental effects were more complicated but modest (i.e. <10% of the variance at all progesterone levels).

Sensitivity analyses within cycle phase again highlighted the importance of genetic factors. Increases in genetic influences were observed in the full models (see online Supplementary Fig. S1), and genetic moderators were retained in most of the best-fitting models (see online Supplementary Table S3). Changes in genetic effects were similar to those across all study days, and genetic moderators were statistically significant in several models (e.g. the follicular, midluteal, and premenstrual phases) (see online Supplementary Table S4).

## Discussion

Using newly adapted twin models that can accommodate repeated measures, we conducted the first study examining daily effects of ovarian hormone levels on genetic/environmental risk for emotional eating. We found novel, non-linear effects of hormones on etiologic risk. Shared environmental influences on emotional eating increased substantially at the highest estradiol levels, while genetic influences were significantly more pronounced at higher progesterone levels. Findings were consistent across primary analyses of all study days and sensitivity analyses within menstrual cycle phase. Taken together, findings significantly advance etiologic models of hormonal risk and provide a new tool for examining the dynamic interplay between risk factors and psychiatric outcomes in intensive longitudinal study designs.

Future studies should identify the mechanisms underlying genetic/environmental effects of daily hormone changes on BE. For estrogen, non-linear changes in shared environmental influences were observed in primary and sensitivity analyses, and shared environmental influences were the only etiologic factors that changed across estradiol levels in phase analyses. The prominent influence of fluctuating estradiol on shared environmental effects suggests estrogen influences emotional eating via processes that are common to co-twins, regardless of their degree of genetic similarity. Hormonally mediated shared environmental influences may reflect membrane estrogen receptor activity, which can affect food intake through non-genomic pathways. Membrane receptor activation can produce rapid molecular signals that change the excitability of neurons within seconds to minutes via processes that do not require changes in gene expression (Santollo, Marshall, & Daniels, 2013). Interestingly, rodent studies show that membrane estrogen receptors exhibit non-linear patterns of activation (i.e. minimal activation below physiological levels) (Mermelstein, Becker, & Surmeier, 1996; Micevych, Kuo, & Christensen, 2009) that would be consistent with non-linear increases in shared environmental influences observed in our analyses. Nonetheless, other data suggest these receptors influence food intake through genomic pathways and changes in gene expression (Graves, Hayes, Fan, & Curtis, 2011; Santollo et al., 2013). These later findings raise the novel possibility that estrogen's shared environmental influences do reflect changes in gene expression – changes that do not vary across individuals with different genetic backgrounds. There is evidence that genes regulating estrogen function are strongly evolutionarily conserved, likely due to their vital role in survival and reproduction (Bondesson, Hao, Lin, Williams, & Gustafsson, 2015; Liu, Zhang, Gladwell, & Teng, 2003). Consequently, it may be that estrogen influences emotional eating through the >99% of genes that do not vary across humans (Collins & Mansoura, 2001). These conserved genes are intriguing candidates for future studies examining mechanisms of estrogen influences on BE in women.

Progesterone's influences were strikingly different from those of estrogen. Changes in daily progesterone levels were primarily associated with differential additive genetic influences, with relatively minimal changes in shared environmental factors. Changes in additive genetic influences suggest that progesterone's effects may operate via genetic variants that do vary between individuals. These types of effects are dependent upon genetic background and reflect the fact that twins who share all their genes (i.e. MZ twins) become more similar to each other in their emotional eating scores across increasing progesterone levels than twins who share proportionally fewer genes (i.e. DZ twins). These findings raise the novel possibility that while estrogen may protect against emotional eating in all women via conserved genomic processes, progesterone may increase risk by causing differential expression of risk and/or protective genes across women who vary in genetic vulnerability to BE. Future studies are needed to directly examine these possibilities and identify the genes and neural systems involved in hormone effects.

One final implication of our results deserves note. Our findings suggest the heritability of BE is not static, but changes across hormone levels and menstrual cycle phase in women. Twin and molecular studies of BE in women should be aware of these changing genetic influences, as genetic ‘signals’ may be stronger or weaker depending on hormone levels and cycle phase. Studies wishing to maximize genetic signals would do well to assess women during hormonal milieus characterized by peak progesterone levels (e.g. the mid-luteal phase). Neuroimaging studies often control for menstrual cycle phase in their study designs (see Sacher, Okon-Singer, & Villringer, 2013), and it may be time for twin/genetic studies to follow suit. Likewise, the mantra that shared environmental influences are not important for BE (and other psychiatric phenotypes) in adulthood in women needs to be modified. Processes that manifest as shared environmental influences in twin models appear to be critically important during peak estradiol levels that correspond to cycle phases also associated with very low progesterone levels (i.e. ovulation). These dynamic changes in estrogen/progesterone levels and etiologic influences represent unique challenges for risk studies of BE but also unique opportunities to alter our study designs and maximize our ability to detect multiple mechanisms that may contribute to BE in women.

Although our study had many strengths (e.g. daily study across 45 days), some limitations should be noted. Our sample was larger than past studies examining ovarian hormone influences on behavior (e.g. Roberts, Eisenlohr-Moul, & Martel, 2018), but our power was still limited for providing precise parameter estimates and examining estrogen × progesterone interactions. Additional studies with still larger samples are needed to fill these gaps. Given significant non-linearity in our models, these studies should explore alternative methods for examining non-linear effects in two-moderator twin models.

Our sample was broadly representative of Michigan in terms of race, ethnicity, and socioeconomic status, but the racial and ethnic diversity of our sample was still limited, and socioeconomic status tended toward more advantaged participants. It will be important to examine hormone effects in more diverse samples (Burnette, Burt, & Klump, 2024), particularly given data suggesting economic and neighborhood disadvantage shifts the curve of genetic risk for disordered eating during key reproductive milestones (e.g. Mikhail et al., 2021).

Our analyses focused on a dimensional measure of BE in a community sample. Although this maximized statistical power, it is unclear whether our findings generalize to clinical BE or binge-related disorders. Past studies show similar phenotypic associations between ovarian hormones, emotional eating, and clinical BE (Klump et al., 2015). Nonetheless, additional studies are needed to replicate and extend our results across the full spectrum of BE severity.

## Supporting information

Klump et al. supplementary material Klump et al. supplementary material

## Data Availability

The data that support the findings of this study are available from the corresponding author, KLK, upon reasonable request. All scripts for the hormone moderation models can be found on the Open Science Framework website at: https://osf.io/cxmuz/?view_only=f41ed357a26049aa9a832293561702ee.

## Footnotes

† The notes appear after the main text.

## Note

1 Notably, our models rest on the assumption that hormones (moderators) are genetically independent of emotional eating (dependent variable). If there is not independence, then genetic mediation (i.e., gene–environment correlations [rGE]) may be present, such that the same genes influence hormone levels and emotional eating. All genetic/environmental covariance between emotional eating and estradiol (Satorra–Bentler χ^2^Δ (df) = .717 (3), *p* = .869) and progesterone (Satorra–Bentler χ^2^Δ (df) = .702 (3), *p* = .873) could be constrained to zero, suggesting no etiologic mediation between hormones and emotional eating.

## Supplementary material

The supplementary material for this article can be found at https://doi.org/10.1017/S0033291724002770
