# Supplementary material for: Daily estradiol and progesterone levels moderate genetic and environmental influences on emotional eating across 45 consecutive days in female twins
Source: Psychol Med. 2024 Dec 4;54(15):4438–46. doi: 10.1017/S0033291724002770 (PMC11650152; doi:10.1017/S0033291724002770)
Supplement: Klump et al. supplementary material [file S0033291724002770sup001.docx]

**Supplemental Material**

Table S1. Hormone and Emotional Eating Descriptive Statistics (N = 468 twins).

| **Variable** | **Sample Sizes** | | | | **Mean (SD)** | **Range** |
| --- | --- | --- | --- | --- | --- | --- |
|  | **Total Observations** | **MZ Twins from Complete Pairs (N)** | **DZ Twins from Complete Pairs (N)** | **Twins without Cotwin Data** |  |  |
| Emotional eating |  |  |  |  |  |  |
| *Included in daily analyses* | 10,555 | 248 | 198 | 0 | 0.30 (.45) | 0-3.69 |
| *Mean follicular phase* | 424 | 216 | 164 | 44 | 0.31 (.39) | 0-2.97 |
| *Mean ovulatory phase* | 433 | 224 | 172 | 37 | 0.29 (.40) | 0-3.10 |
| *Mean midluteal phase* | 439 | 230 | 174 | 35 | 0.32 (.41) | 0-3.01 |
| *Mean premenstrual phase* | 454 | 232 | 192 | 30 | 0.30 (.43) | 0-3.23 |
| *Mean menstrual phase* | 466 | 250 | 200 | 16 | 0.30 (.43) | 0-3.15 |
|  |  |  |  |  |  |  |
| Estradiol (pg/ml) |  |  |  |  |  |  |
| *Included in daily analyses* | 10,618 | 248 | 198 | 0 | 2.95 (1.53) | 0.22-14.38 |
| *Mean follicular phase* | 414 | 204 | 160 | 50 | 2.60 (1.30) | 0.54-11.78 |
| *Mean ovulatory phase* | 434 | 226 | 172 | 36 | 3.36 (1.58) | 0.29-11.93 |
| *Mean midluteal phase* | 438 | 230 | 172 | 36 | 3.09 (1.56) | 0.62-13.10 |
| *Mean premenstrual phase* | 455 | 238 | 190 | 27 | 2.67 (2.53) | 0.21-47.62 |
| *Mean menstrual phase* | 423 | 214 | 162 | 47 | 2.54 (1.45) | 0.26-12.57 |
|  |  |  |  |  |  |  |
| Progesterone (pg/ml) |  |  |  |  |  |  |
| *Included in daily analyses* | 10,618 | 248 | 198 | 0 | 124.15 (88.27) | 6.80-648.63 |
| *Mean follicular phase* | 418 | 208 | 162 | 48 | 81.81 (53.95) | 11.74-336.42 |
| *Mean ovulatory phase* | 434 | 226 | 172 | 36 | 99.34 (65.12) | 11.16-559.61 |
| *Mean midluteal phase* | 439 | 230 | 174 | 35 | 179.75 (95.04) | 20.70-517.54 |
| *Mean premenstrual phase* | 458 | 236 | 196 | 26 | 112.73 (69.42) | 17.24-410.01 |
| *Mean menstrual phase* | 436 | 220 | 178 | 38 | 92.06 (61.72) | 11.48-449.25 |

***Note.*** SD = standard deviation. Values for individual phases represent mean values for each participant during study days in that phase (e.g., the mean value of emotional eating across days in the follicular phase). Included participants had complete hormone and emotional eating data on at least 13 study days (mean = 34.10, *SD* = 6.44), with >90% of study participants having complete data for at least 25 days.

**Sensitivity Analyses within Menstrual Cycle Phase**

***Menstrual Phase Coding***

Menstrual cycle phases (i.e., follicular, ovulatory, mid-luteal, premenstrual, menstrual) and anovulation were coded by trained raters using daily hormone values and dates of menstrual bleeding via procedures described previously (see Klump et al., 2015). All raters underwent extensive training, and each rater had to achieve an inter-rater reliability of ≥.80.

***Twin Models***

Hormone and emotional eating values for a particular participant were averaged across all days within each phase (e.g., all follicular phase days), yielding one value for each participant for each phase. We focused on these average levels given the highly variable (e.g., 1-14 days) and in some cases, very small number of observations (e.g., the ovulatory phase ~1-3 days) in each phase. Further, because of the way the daily analyses were structured, twins would have needed to be in the same phase on a given day of study participation for that day’s data to be included in the model. Relying on individual daily values for phase analyses therefore would have led to a substantial amount of missing data. We log-transformed emotional eating scores to account for positive skew, as these analyses used the traditional maximum likelihood estimator that is less robust to non-normality than the robust FIML estimator used in daily analyses. Hormone values were “binned” (into deciles – given the smaller range of values within phase) across all participants for a particular phase to ensure multiple observations at each moderator level. Because these analyses were within phase, these models did not control for study day or menstruation, but were otherwise identical to those depicted in Figure 1.

**Table S2.** Model Fit Comparisons for Estrogen Models within Menstrual Cycle Phases.

| **Models** | **-2lnL** | **χ^2^Δ (df)** | ***p*** | **AIC** | **BIC** | **SABIC** |
| --- | --- | --- | --- | --- | --- | --- |
| **Follicular Phase** | | | | | | |
| Full model | 14.19 | — | — | 44.19 | 92.25 | 44.74 |
| No A mods | 18.18 | 3.99 (4) | .407 | 40.18 | 75.43 | 40.59 |
| No C mods |  |  |  |  |  |  |
| No E mods | 17.94 | 3.75 (4) | .441 | 39.94 | 75.19 | 40.35 |
| No A and C mods | 17.28 | 3.09 (2) | .214 | 43.28 | 84.93 | 43.76 |
| No A and E mods | Estimation issue | | | | | |
| No C and E mods | 17.41 | 3.22 (2) | .200 | 43.41 | 85.06 | 43.89 |
| **No moderation** | **18.83** | **4.64 (6)** | **.590** | **36.83** | **65.67** | **37.17** |
| **Ovulatory Phase** |  |  |  |  |  |  |
| Full model | .52 | — | — | 30.52 | 79.92 | 32.40 |
| No A mods | .65 | .14 (2) | .934 | 26.65 | 69.47 | 28.28 |
| No C mods | 2.18 | 1.67 (2) | .434 | 28.18 | 71.00 | 29.81 |
| No E mods | .64 | .12 (2) | .942 | 26.64 | 69.45 | 28.26 |
| No AE mods | .72 | .20 (4) | .995 | 22.72 | 58.95 | 24.10 |
| No AE mods, *no C quad mod* | 2.79 | 2.28 (5) | .809 | 22.79 | 55.73 | 24.05 |
| No AE mods, *no C linear mod* | 1.16 | .65 (5) | .986 | 21.16 | 54.10 | 22.41 |
| **No AE mods, *no C linear mod, no C main effect*** | **1.17** | **.65 (6)** | **.995** | **19.17** | **48.81** | **20.30** |
| No moderation | 10.75 | 10.23 (6) | .115 | 28.75 | 58.39 | 29.88 |
| **Midluteal Phase** |  |  |  |  |  |  |
| Full model | 12.19 | — | — | 42.19 | 91.74 | 44.22 |
| No A mods | 28.73 | 16.54 (2) | <.001 | 54.73 | 97.68 | 56.49 |
| No C mods | 22.65 | 10.46 (2) | .005 | 48.65 | 91.59 | 50.41 |
| No E mods | 13.25 | 1.05 (2) | .590 | 39.25 | 82.19 | 41.00 |
| No E mods, *no A quad mod* | 13.65 | 1.46 (3) | .692 | 37.65 | 77.29 | 39.27 |
| **No AE mods** | **13.72** | **1.53 (4)** | **.822** | **35.72** | **72.05** | **37.21** |
| No AE mods, *no C linear mod* | 17.74 | 5.54 (5) | .353 | 37.74 | 70.77 | 39.09 |
| No AE mods, *no C quad mod* | 16.07 | 3.88 (5) | .567 | 36.07 | 69.11 | 37.43 |
| No moderation | 18.58 | 6.39 (6) | .381 | 36.58 | 66.31 | 37.80 |
| **Premenstrual Phase** |  |  |  |  |  |  |
| Full model | 58.92 | — | — | 88.92 | 139.27 | 91.74 |
| No A mods | 59.24 | .32 (2) | .852 | 85.24 | 128.88 | 87.68 |
| No C mods | 60.96 | 2.04 (2) | .360 | 86.96 | 130.60 | 89.40 |
| No E mods | 59.93 | 1.01 (2) | .605 | 85.93 | 129.56 | 88.37 |
| No AE mods | 60.71 | 1.79 (4) | .774 | 82.71 | 119.64 | 84.78 |
| No AE mods, *no C main effect* | 60.86 | 1.94 (5) | .857 | 80.86 | 114.43 | 82.74 |
| No AE mods, *no C linear mod* | 61.37 | 2.45 (5) | .784 | 81.37 | 114.93 | 83.25 |
| **No AE mods, *no C linear mod, no C main effect*** | **61.55** | **2.63 (6)** | **.854** | **79.55** | **109.76** | **81.24** |
| No moderation | 64.52 | 5.60 (6) | .469 | 82.52 | 112.73 | 84.22 |
| **Menstrual Phase** |  |  |  |  |  |  |
| Full model | 22.72 | — | — | 52.72 | 101.18 | 53.67 |
| No A mods | 24.39 | 1.67 (2) | .947 | 50.39 | 92.39 | 51.22 |
| No A quad mod | 23.56 | .85 (1) | .358 | 51.56 | 96.80 | 52.45 |
| No A linear mod | 23.16 | .44 (1) | .507 | 51.16 | 96.39 | 52.05 |
| No C mods | 93.93 | 71.21 (2) | <.001 | 119.93 | 161.93 | 120.76 |
| No C quad mod | 68.21 | 45.50 (1) | <.001 | 96.21 | 141.45 | 97.10 |
| No C linear mod | 22.79 | .08 (1) | .783 | 50.79 | 96.03 | 51.69 |
| No E mods | 23.81 | 1.09 (2) | .579 | 49.81 | 91.82 | 50.64 |
| No E quad mod | 23.21 | .49 (1) | .484 | 51.21 | 96.44 | 52.10 |
| No E linear mod | 22.94 | .22 (1) | .639 | 50.94 | 96.17 | 51.83 |
| No AE mods | 24.66 | 1.94 (4) | .746 | 46.66 | 82.20 | 47.36 |
| No AE mods, *no C main effect* | 25.57 | 2.85 (5) | .723 | 45.57 | 77.88 | 46.20 |
| No AE mods, *no C linear mod* | 25.92 | 3.21 (5) | .668 | 45.92 | 78.23 | 46.56 |
| **No AE mods, *no C linear mod, no C main effect*** | **25.92** | **3.21 (6)** | **.783** | **43.92** | **73.00** | **44.50** |
| No moderation | 30.23 | 7.51 (6) | .276 | 48.23 | 77.31 | 48.80 |

Note. A = additive genetic influences, C = shared environmental influences, E = nonshared environmental influences; quad = quadratic/non-linear effects; -2lnL = minus twice the log-likelihood; ∆χ^2^ = the difference in -2lnL values between the full model and the nested model, which is chi-square distributed under the null hypothesis implied by the reduced model; df = degrees of freedom; AIC = Akaike Information Criterion; BIC = Bayesian Information Criterion; SABIC = sample size adjusted Bayesian Information Criterion. Dashes indicate parameters are not applicable. The best-fitting model is bolded.

**Table S3.** Model Fit Comparisons for Progesterone Models within Menstrual Cycle Phases.

| **Models** | **-2lnL** | **χ^2^Δ (df)** | ***p*** | **AIC** | **BIC** | **SABIC** |
| --- | --- | --- | --- | --- | --- | --- |
| **Follicular Phase** | | | | | | |
| Full model | 9.19 | — | — | 39.19 | 87.25 | 39.74 |
| No A mods | 98.63 | 89.45 (2) | <.001 | 124.63 | 166.29 | 125.11 |
| No A quad mod | 9.80 | .61 (1) | .435 | 37.80 | 82.65 | 38.31 |
| **No A quad mod, *no C quad mod, no E quad mod, no A main effect*** | **11.25** | **2.06 (4)** | **.724** | **33.25** | **68.50** | **33.66** |
| No C mods | 72.02 | 62.84 (2) | <.001 | 98.02 | 139.68 | 98.50 |
| No C quad mod | 9.27 | .09 (1) | .767 | 37.28 | 82.13 | 37.79 |
| No E mods | 12.62 | 3.44 (2) | .179 | 38.62 | 80.27 | 39.10 |
| No E quad mod | 9.71 | .52 (1) | .471 | 37.71 | 82.56 | 38.22 |
| No moderation | 19.98 | 10.79 (6) | .095 | 37.98 | 66.81 | 38.31 |
|  |  |  |  |  |  |  |
| **Ovulatory Phase** |  |  |  |  |  |  |
| Full model | -2.27 | — | — | 27.73 | 77.13 | 29.61 |
| No A mods | -1.77 | .50 (2) | .777 | 24.23 | 67.04 | 25.86 |
| No C mods | -2.26 | .01 (2) | .993 | 23.74 | 66.55 | 25.37 |
| No E mods | 6.88 | 9.15 (2) | .010 | 32.88 | 75.69 | 34.50 |
| No E quad mod | -1.90 | .37 (1) | .541 | 26.10 | 72.21 | 27.85 |
| No E linear mod | -2.23 | .04 (1) | .834 | 25.77 | 71.88 | 27.52 |
| No AC mods | -1.60 | .68 (4) | .954 | 20.40 | 56.63 | 21.78 |
| No AC mods, *no E linear mod* | -1.40 | .87 (5) | .972 | 18.60 | 51.53 | 19.85 |
| **No AC mods, *no E linear mod, no C main effect*** | **-1.40** | **.87 (6)** | **.990** | **16.60** | **46.24** | **17.72** |
| No moderation | 10.62 | 12.89 (6) | .045 | 28.62 | 58.26 | 29.74 |
| **Midluteal Phase** |  |  |  |  |  |  |
| Full model | -14.73 | — | — | 15.27 | 64.82 | 17.30 |
| No A mods | -11.15 | 3.58 (2) | .167 | 14.85 | 57.79 | 16.61 |
| No C mods | -4.77 | 9.96 (2) | .007 | 21.23 | 64.17 | 22.98 |
| No E mods | -7.50 | 7.23 (2) | .027 | 18.50 | 61.44 | 20.25 |
| No E linear mod | -14.42 | .31 (1) | .576 | 13.58 | 59.83 | 15.47 |
| No E quad mod | -14.67 | .07 (1) | .797 | 13.33 | 59.58 | 15.23 |
| **No E quad mod, *no A main effect*** | **-14.53** | **.20 (2)** | **.906** | **11.47** | **54.41** | **13.22** |
| No E quad mod, *no A mods* | -10.64 | 4.09 (3) | .251 | 13.36 | 53.00 | 14.99 |
| No E quad mod, *no A mods, no A main effect* | -.28 | 14.46 (4) | .006 | 21.72 | 58.06 | 23.21 |
| No moderation | 20.55 | 35.28 (6) | <.001 | 38.55 | 68.28 | 39.76 |
| **Premenstrual Phase** |  |  |  |  |  |  |
| Full model | 47.38 | — | — | 77.38 | 127.73 | 80.20 |
| No A mods | 49.48 | 2.10 (2) | .350 | 75.48 | 119.11 | 77.92 |
| No A quad mod | 47.74 | .36 (1) | .550 | 75.74 | 122.73 | 78.37 |
| No A linear mod | 47.44 | .06 (1) | .880 | 75.44 | 122.43 | 78.07 |
| No A mods, *no C quad mod* | 49.79 | 2.41 (3) | .491 | 73.79 | 114.07 | 76.05 |
| **No A mods, *no C quad mod, no E linear mod*** | **50.11** | **2.73 (4)** | **.604** | **72.11** | **109.03** | **74.17** |
| No C mods | 54.58 | 7.20 (2) | .027 | 80.58 | 124.21 | 83.02 |
| No C quad mod | 48.20 | .83 (1) | .363 | 76.20 | 123.20 | 78.84 |
| No C linear mod | 48.42 | 1.04 (1) | .307 | 76.42 | 123.41 | 79.05 |
| No E mods | 51.62 | 4.25 (2) | .120 | 77.62 | 121.26 | 80.07 |
| No E quad mod | 49.85 | 2.47 (1) | .116 | 77.85 | 124.84 | 80.48 |
| No E linear mod | 48.64 | 1.26 (1) | .262 | 76.64 | 123.63 | 79.27 |
| No AE mods | 51.67 | 4.29 (4) | .368 | 73.67 | 110.59 | 75.74 |
| No AE mods, *no C quad mod* | 52.79 | 5.42 (5) | .367 | 72.79 | 106.36 | 74.67 |
| No moderation | 59.31 | 11.93 (6) | .064 | 77.31 | 107.52 | 79.00 |
| **Menstrual Phase** |  |  |  |  |  |  |
| Full model | 21.83 | — | — | 51.83 | 100.30 | 52.79 |
| No A mods | 25.84 | 4.01 (2) | .135 | 51.84 | 93.85 | 52.67 |
| No A quad mod | 24.80 | 2.97 (1) | .085 | 52.81 | 98.04 | 53.70 |
| No A linear mod | 23.82 | 1.99 (1) | .159 | 51.82 | 97.06 | 52.71 |
| No C mods | 34.71 | 12.87 (2) | .002 | 60.71 | 102.71 | 61.53 |
| No C quad mod | 21.85 | .01 (1) | .906 | 49.85 | 95.08 | 50.74 |
| No C linear mod | 21.85 | .01 (1) | .906 | 49.85 | 95.08 | 50.74 |
| **No C mods, *no C main effect*** | **21.85** | **.01 (3)** | **1.000** | **45.85** | **84.62** | **46.61** |
| No AC mods, no C main effect | 29.07 | 7.24 (5) | .203 | 49.07 | 81.39 | 49.71 |
| No E mods | 28.00 | 6.17 (2) | .046 | 54.01 | 96.01 | 54.83 |
| No moderation | 30.11 | 8.27 (6) | .219 | 48.11 | 77.19 | 48.68 |

Note. A = additive genetic influences, C = shared environmental influences, E = nonshared environmental influences; quad = quadratic/non-linear effects; -2lnL = minus twice the log-likelihood; **χ^2^Δ** = chi square change; df = degrees of freedom; AIC = Akaike Information Criterion; BIC = Bayesian Information Criterion; SABIC = sample size adjusted Bayesian Information Criterion. Dashes indicate parameters are not applicable. The best-fitting model is bolded.

**Table S4.** Parameter Estimates from the Full and Best-Fitting Models for Estrogen and Progesterone within Menstrual Cycle Phase.

| Models | **a** | | **Linear**  **a mod** | **Quad**  **a mod** | **c** | | **Linear**  **c mod** | | **Quad**  **c mod** | | | **e** | **Linear**  **e mod** | | **Quad**  **e mod** |
| --- | --- | --- | --- | --- | --- | --- | --- | --- | --- | --- | --- | --- | --- | --- | --- |
| **Estrogen Models** | | | | | | | | | | | | | | | |
| ***Follicular Phase*** | | |  |  |  | |  | |  | | |  |  | |  |
| Full model | **-.21**  **(-.31, -.10)** | | .43  (-.10, .97) | -.30  (-.87, .26) | .05  (-.16, .26) | | -.02  (-.69, .65) | | .14  (-.44, .72) | | | **.14**  **(.07, .21)** | .30  (-.01, .62) | | -.26  (-.57, .05) |
| Best fitting | .09  (-.10, .28) | | — | — | .09  (-.07, .26) | | — | | — | | | **.22**  **(.19, .24)** | — | | — |
| ***Ovulatory Phase*** | | | | | | | | | | | | | | | |
| Full model | | **-.10**  **(-.21, -.001)** | -.09  (-.59, .40) | .11  (-.50, .73) | .04  (-.12, .19) | | -.19  (-.78, .41) | .38  (-.21, .97) | | **.20**  **(.14, .26)** | | | -.03  (-.31, .25) | | .04  (-.24, .32) |
| Best fitting | | **-.11**  **(-.16, -.06)** | — | — | — | | — | **.21**  **(.13, .30)** | | **.20**  **(.18, .23)** | | | — | | — |
| ***Midluteal Phase*** | | |  |  |  | |  |  | |  | | |  | |  |
| Full model | | **-.17**  **(-.32, -.02)** | .05  (-.72, .81) | .01  (-.67, .68) | -.12  (-.33, .08) | | .60  (-.03, 1.22) | -.35  (-.91, .21) | | **.21**  **(.13, .29)** | | | -.17  (-.53, .19) | | .17  (-.17, .51) |
| Best fitting | | **.15**  **(.11, .19)** | — | — | **-.17**  **(-.27, -.07)** | | **.67**  **(.19, 1.15)** | -.39  (-.84, .06) | | **.18**  **(.16, .21)** | | | — | | — |
| ***Premenstrual Phase*** | | |  |  |  | |  |  | |  | | |  | |  |
| Full model | | **-.15**  **(-.25, -.05)** | -.04  (-.55, .47) | .10  (-.46, .66) | .05  (-.11, .20) | | -.25  (-.81, .32) | .43  (-.07, .94) | | **.22**  **(.16, .28)** | | | -.02  (-.31, .27) | | -.02  (-.30, .27) |
| Best fitting | | **-.15**  **(-.20, -.11)** | — | — | — | | — | **.16**  **(.06, .26)** | | **.21**  **(.18, .23)** | | | — | | — |
| ***Menstrual Phase*** | |  |  |  |  | |  |  | |  | | |  | |  |
| Full model | | **.17**  **(.08, .27)** | -.19  (-.74, .35) | .28  (-.30, .85) | .04  (-.18, .25) | | -.21  (-1.16, .74) | .27  (-.79, 1.34) | | **.19**  **(.13, .25)** | | | .07  (-.23, .38) | | -.11  (-.42, .20) |
| Best fitting | | **-.17**  **(-.21, -.12)** | — | — | — | | — | **.17**  **(.08, .26)** | | **.19**  **(.16, .21)** | | | — | | — |
| **Progesterone Models** | | | | | | | | | | | | | | | |
| ***Follicular Phase*** | |  |  |  | |  |  |  | | |  | |  |  | |
| Full model | | .02  (-.17, .22) | -.46  (-1.21, .28) | .31  (-.46, 1.08) | | **-.20**  **(-.30, -.10)** | .27  (-.26, .79) | .09  (-.44, .61) | | | **.21**  **(.13, .28)** | | .06  (-.32, .43) | -.13  (-.47, .22) | |
| Best fitting | | — | **.20**  **(.13, .26)** | — | | **.18**  **(.09, .27)** | **-.21**  **(-.40, -.03)** | — | | | **.24**  **(.19, .29)** | | -.08  (-.17, .01) | — | |
| ***Ovulatory Phase*** | |  |  |  | |  |  |  | | |  | |  |  | |
| Full model | | -.13  (-.35, .10) | -.02  (-.67, .63) | -.03  (-.54, .49) | | .07  (-.44, .57) | -.13  (-1.28, 1.02) | .10  (-.88, 1.07) | | | **.25**  **(.18, .31)** | | -.03  (-.34, .28) | -.09  (-.37, .19) | |
| Best fitting | | **.15**  **(.12, .19)** | — | — | | — | — | — | | | **.23**  **(.20, .26)** | | — | **-.10**  **(-.16, -.05)** | |
| ***Midluteal Phase*** | |  |  |  | |  |  |  | | |  | |  |  | |
| Full model | | .05  (-.22, .32) | -.71  (-1.77, .34) | .56  (-.41, 1.53) | | **.33**  **(.23, .43)** | **-1.40**  **(-1.98,**  **-.82)** | **1.36**  **(.87, 1.86)** | | | **.21**  **(.15, .26)** | | -.07  (-.33, .18) | -.03  (-.26, .20) | |
| Best fitting | | — | **.52**  **(.31, .73)** | **-.39**  **(-.68, -.09)** | | **.33**  **(.23, .43)** | **-1.45**  **(-1.94,**  **-.96)** | **1.40**  **(.96, 1.84)** | | | **.21**  **(.17, .25)** | | **-.11**  **(-.17, -.04)** | — | |
| ***Premenstrual Phase*** | | |  |  | |  |  |  | | |  | |  |  | |
| Full model | | -.13  (-.40, .13) | .10  (-.68, .88) | -.18  (-.75, .40) | | **-.23**  **(-.40, -.06)** | .56  (-.15, 1.26) | -.35  (-1.15, .45) | | | **.19**  **(.11, .26)** | | .19  (-.12, .51) | -.25  (-.53, .04) | |
| Best fitting | | **.16**  **(.12, .20)** | — | — | | **-.17**  **(-.26, -.07)** | **.26**  **(.10, .43)** | — | | | **.22**  **(.18, .25)** | | -.06  (-.13, .01) | — | |
| ***Menstrual Phase*** | |  |  |  | |  |  |  | | |  | |  |  | |
| Full model | | **.24**  **(.10, .38)** | -.41  (-.96, .14) | .45  (-.04, .93) | | .06  (-.45, .57) | -.19  (-1.81, 1.44) | .16  (-1.25, 1.56) | | | **.13**  **(.07, .19)** | | **.36**  **(.06, .67)** | **-.39**  **(-.67, -.10)** | |
| Best fitting | | **.24**  **(.18, .31)** | **-.43**  **(-.78, -.08)** | **.47**  **(.12, .81)** | | — | — | — | | | **.13**  **(.07, .19)** | | **.36**  **(.06, .67)** | **-.39**  **(-.67, -.10)** | |

Note. a = additive genetic influences; c = shared environmental influences; e = nonshared environmental influences; mod = moderator; quad = quadratic/non-linear effects. Dashes indicate that the parameter estimate was constrained to be 0. Significant model parameters are bolded, with the 95% CI in parentheses.

| **Estrogen** | |
| --- | --- |
| ***Follicular Phase*** | |
| ***Full Model*** | ***Best-Fitting*** |
| 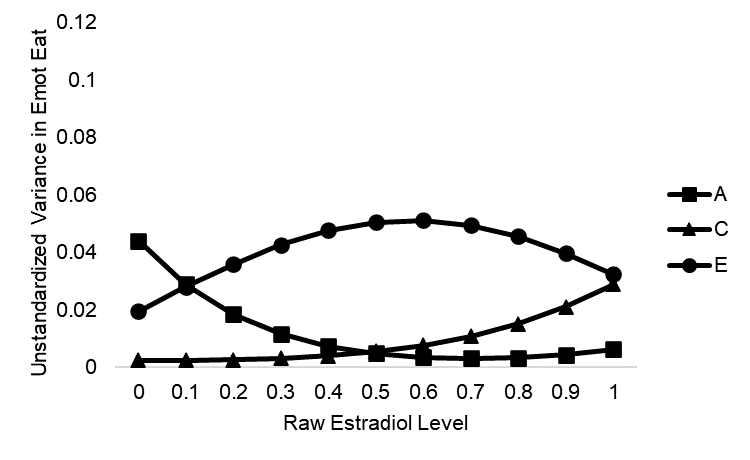 | 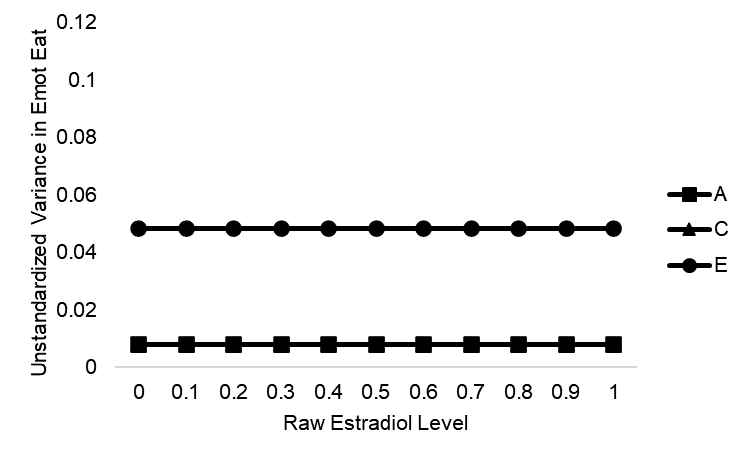 |

| ***Ovulatory Phase*** | |
| --- | --- |
| ***Full Model*** | ***Best-Fitting*** |
| 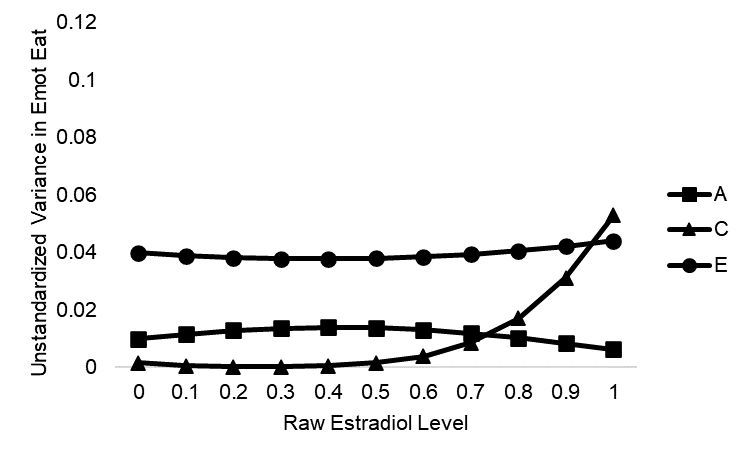 | 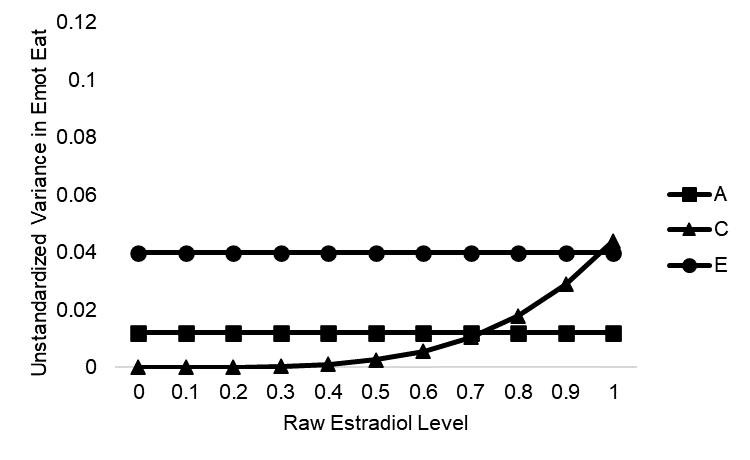 |
| ***Midluteal Phase*** | |
| ***Full Model*** | ***Best-Fitting*** |
| 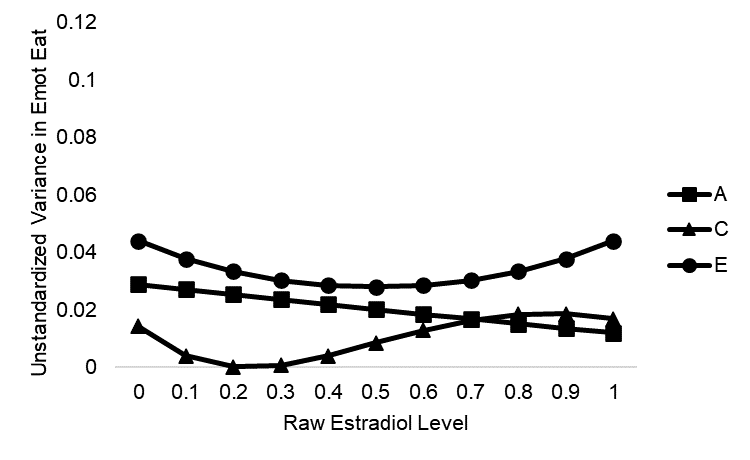 | 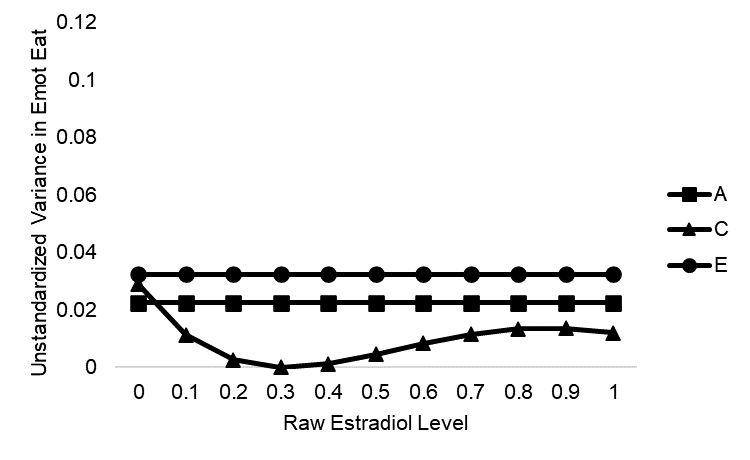 |

| ***Premenstrual Phase*** | |
| --- | --- |
| ***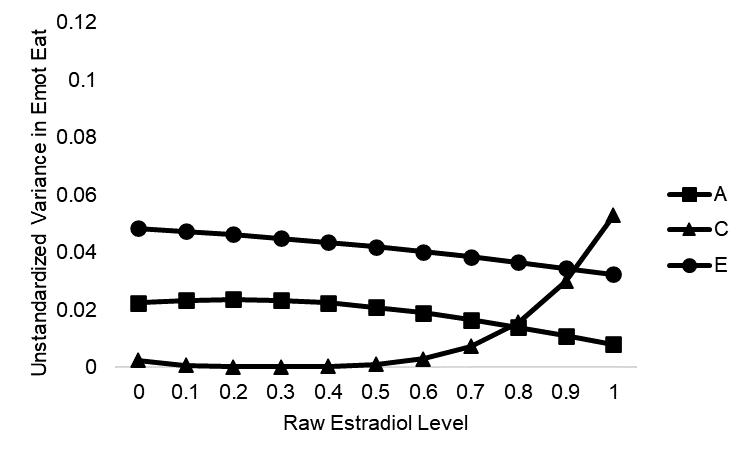Full Model*** | ***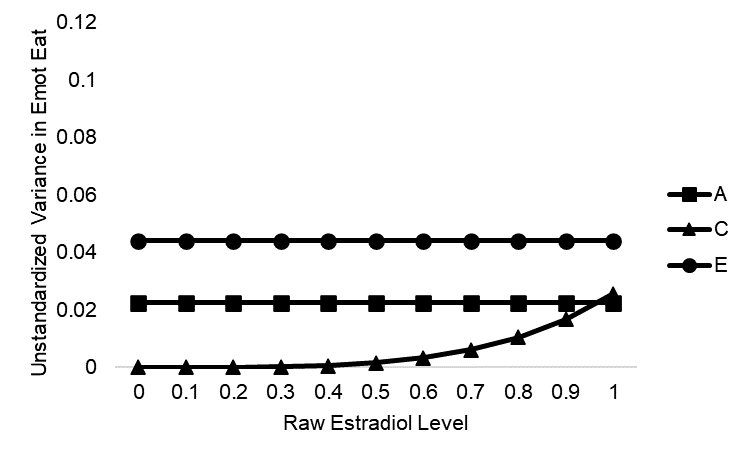Best-Fitting*** |
|  |  |
| ***Menstrual Phase*** | |
| ***Full Model*** | ***Best-Fitting*** |
| 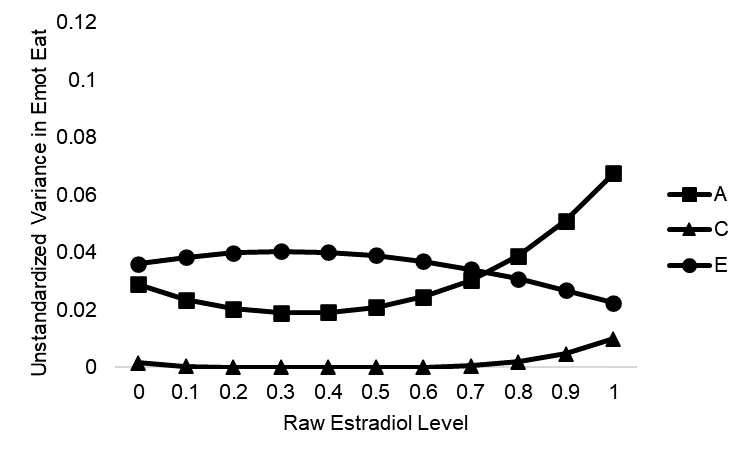 | 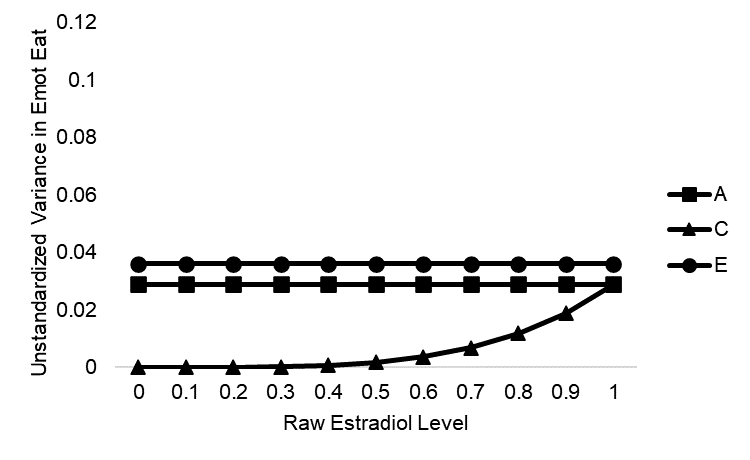 |

| **Progesterone** | |
| --- | --- |
| ***Follicular Phase*** | |
| ***Full Model*** | ***Best-Fitting*** |
| 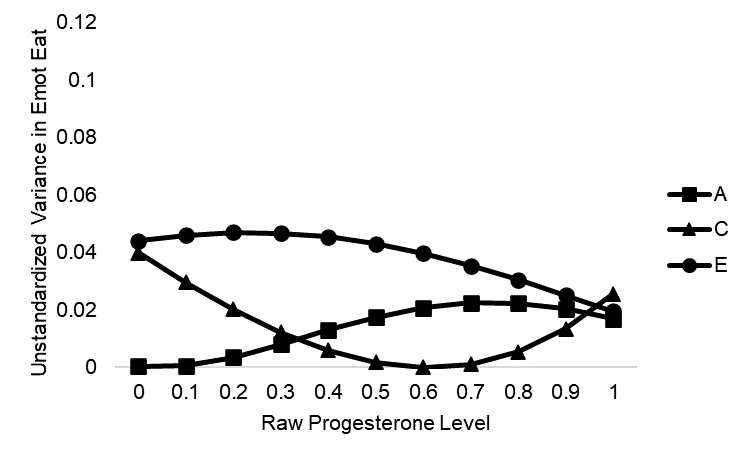 | 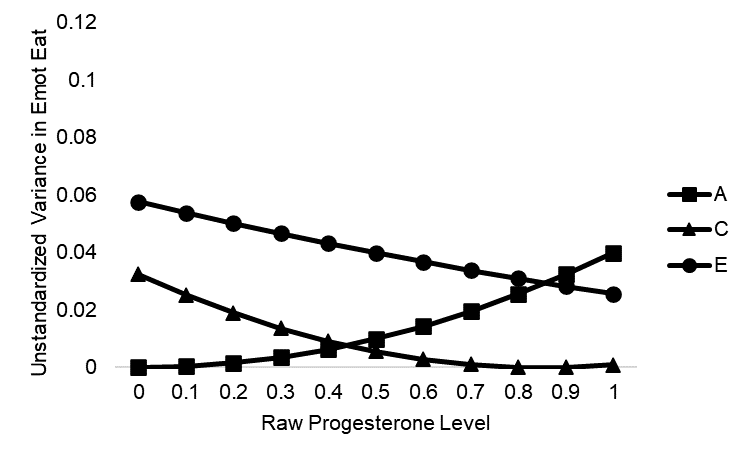 |

| ***Ovulatory Phase*** | |
| --- | --- |
| ***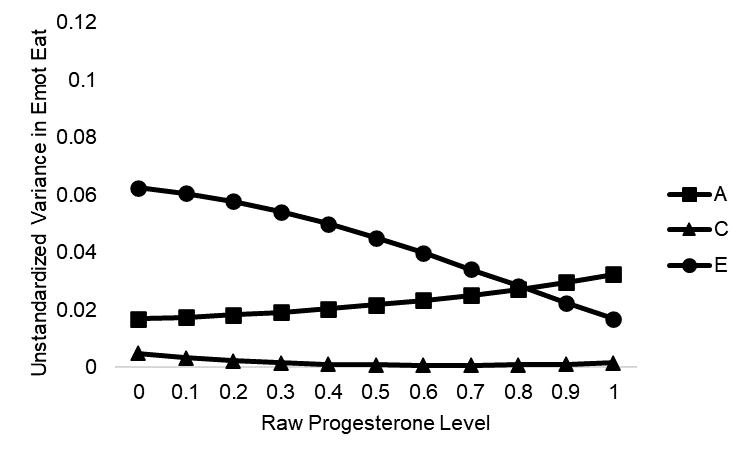Full Model*** | ***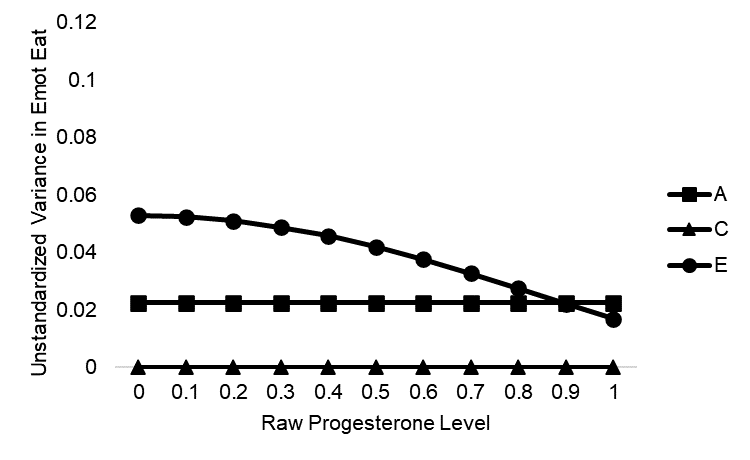Best-Fitting*** |
|  |  |
| ***Midluteal Phase*** | |
| ***Full Model*** | ***Best-Fitting*** |
| 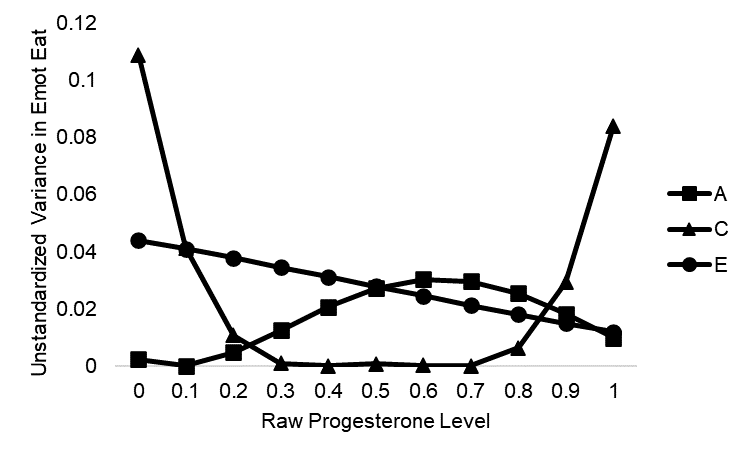 | 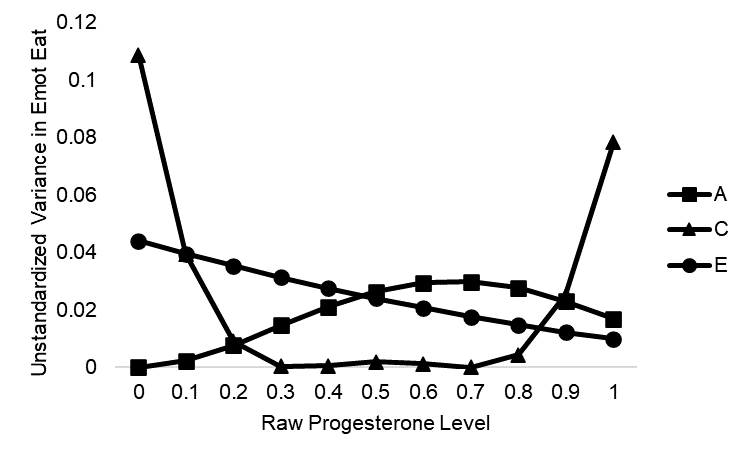 |

| ***Premenstrual Phase*** | |
| --- | --- |
| ***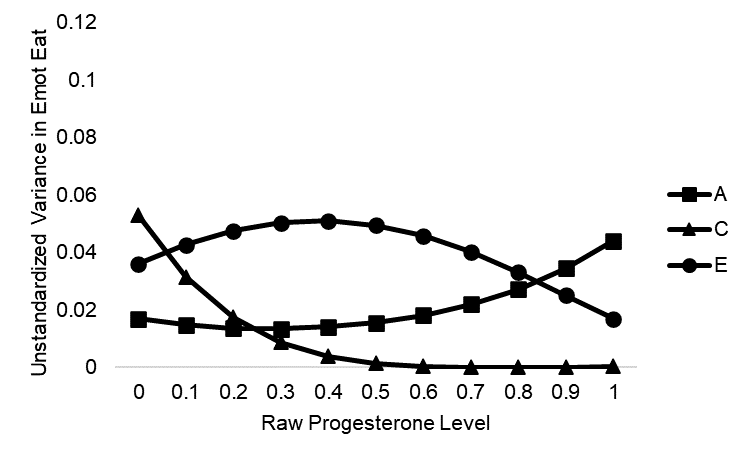Full Model*** | ***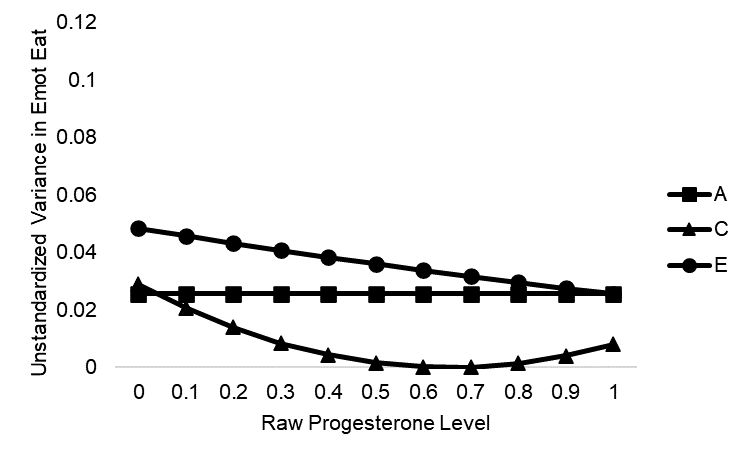Best-Fitting*** |
|  |  |
| ***Menstrual Phase*** | |
| ***Full Model*** | ***Best-Fitting*** |
| 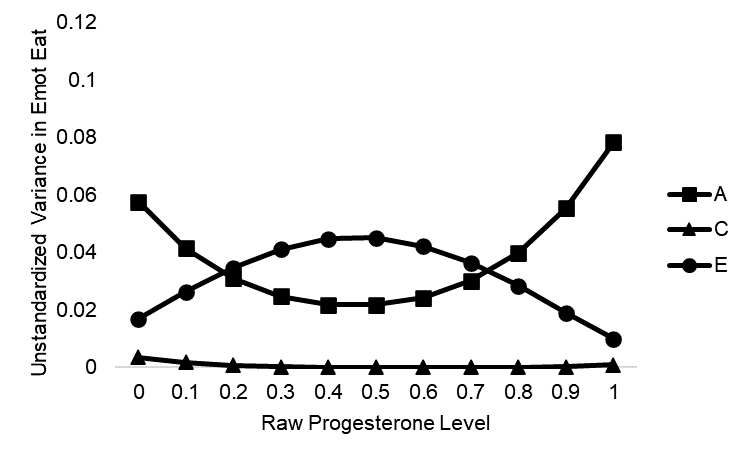 | 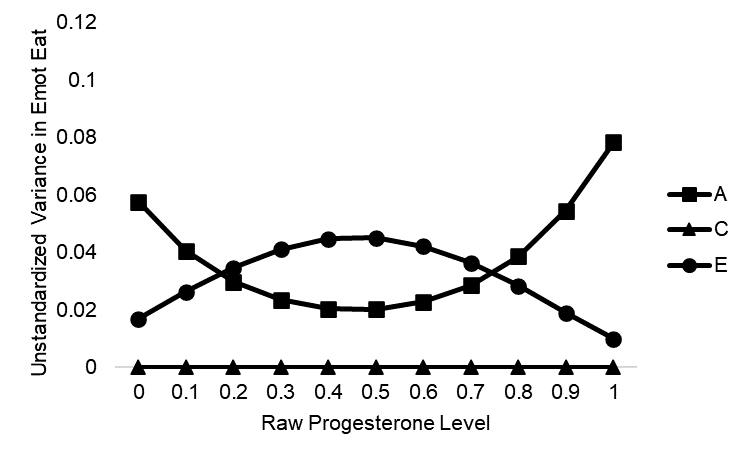 |

Figure S1. Changes in Genetic and Environmental Influences on Emotional Eating across Ovarian Hormone Levels in the Full and Best-Fitting Models. A = additive genetic influences; C = shared environmental influences; E = nonshared environmental influences; Emot Eat = emotional eating. The X axis depicts raw hormone values that were binned into deciles for analyses.
